# Reduction of Pain and Edema of the Legs by Walking Wearing Elastic Stockings

**DOI:** 10.1155/2015/648074

**Published:** 2015-08-23

**Authors:** Carlos Alberto Carvalho, Renata Lopes Pinto, Maria de Fatima Guerreiro Godoy, Jose Maria Pereira de Godoy

**Affiliations:** ^1^Medical School in São José do Rio Preto, FAMERP, Avenida Constituição 1306, 15025-120 São José do Rio Preto, SP, Brazil; ^2^Godoy Clinic Research Group, São José do Rio Preto, SP, Brazil; ^3^Cardiovascular Surgery Department of the Medicine School in São José do Rio Preto, FAMERP and CNPq (National Council for Research and Development), Avenida Constituição 1306, 15025-120 São José do Rio Preto, SP, Brazil

## Abstract

*Aim*. The objective of the current study was to evaluate the reduction of edema and pain with the use of elastic stockings. *Method*. The effect of walking on a treadmill for 50 minutes in the evening wearing elastic compression stockings on pain and edema was evaluated in a prospective randomized crossover clinical trial. In Assessment 1, the legs of participants were measured by volumetry at 7:00 a.m. and they were asked to perform their normal daily activities and to return at 4:00 p.m. Forty-two legs of 21 female patients with ages of the participants ranged from 32 to 72 years with signs and symptoms of chronic venous disease. The sizes of the legs of all patients were evaluated by water displacement volumetry and a visual analog scale was used to assess pain. *Results*. After walking for 50 minutes on the treadmill, the volume reduced (paired *t*-test: *p* value < 0.03). In relation to pain, there was a reduction in pain after the treadmill session using the elastic stocking (Wilcoxon signed rank test: *p* value < 0.007). *Conclusion*. The reduction of edema and pain of the legs during the course of the day can be accomplished with the use of elastic stockings, as well as walking.

## 1. Introduction

Chronic venous disease affects millions of people throughout the world causing signs and symptoms that lead to disablement from work and loss of quality of life [1, 2]. However the symptoms reported by patients are not always exclusively due to venous diseases. Therefore, a differential diagnosis must be made, in particular in respect to orthopedic alterations especially those involving the feet [2].

Another condition that may cause or aggravate the symptoms is the external environment due to gravitational pressure and temperature. It has been reported that individuals working in specific positions may feel leg pain, have a bloated feeling, have a feeling of heaviness, and have several other disturbing sensations [3]. These symptoms can be relieved with the use of elastic stockings and compression with bandages [3–6].

Elastic stockings must be correctly indicated and adjusted to the leg. Artifacts such as folds and pleats can exert a strangling effect. Compression stockings act by exerting a pressure at the stocking-skin interface that is transmitted to the surrounding tissues causing a pressure differential in the cell interstice and arteries, veins, and lymphatics. While, at rest, stockings generate an almost constant pressure called resting pressure, with movement, such as walking, the stockings generate variations in pressure [7].

The use of elastic stockings is well-defined for the treatment of venous and lymphatic disease; however, a careful analysis is required of the benefits of adherence to treatment and unpleasant situations such as feeling hot, irritation, and itching. In tropical countries, such as Brazil, the heat is a limiting factor which can lead to treatment failure in a short period of time [8]. However, flexibility as to the use of stockings during the day may help patient compliance.

One study evaluating the use of stockings during half the day showed that this benefits compliance; even so compression mechanisms should ideally be used during the entire day [6]. Another study showed that there is a reduction of edema when stockings are worn while walking in the evening. The objective of the current study was to evaluate the reduction of edema and pain with the use of elastic stockings.

## 2. Method

The effect of walking on a treadmill for 50 minutes in the evening wearing elastic compression stockings on pain and edema was evaluated in a prospective randomized crossover clinical trial.

Forty-two legs of 21 female patients were evaluated in this study conducted at the Clinica Godoy in 2013/2014. The ages of the participants ranged from 32 to 72 years with a mean of 49.5 years.

Patients with signs and symptoms of chronic venous disease with at least one leg having a Clinical, Etiology, Anatomy and Pathophysiology (CEAP) classification of C3 (swollen ankle (oedema) due to varicose veins or hidden varicose veins-venous reflux) were enrolled in this study. The inclusion criteria were edema and fatigue of the legs which worsened during the day but improved with the rest and with elevation of the legs and pain in the legs. The exclusion criteria were varicose veins with CEAP classifications 4, 5, and 6, difficulty walking, morbid obesity, orthopedic changes, and other diseases clinically evaluated which might cause the symptoms of the legs.

The sizes of the legs of all patients were evaluated by water displacement volumetry and a visual analog scale was used to assess pain.

In Assessment 1, the legs of participants were measured by volumetry at 7:00 a.m. and they were asked to perform their normal daily activities and to return at 4:00 p.m. At 4:00 p.m., the legs were again evaluated by volumetry and patients indicated the pain that they felt using the analog pain scale. After putting on Venosan 20/30 mmHg knee-length elastic compression stockings, participants walked for 50 minutes on a treadmill at a speed of 3 km per hour. They were again assessed by volumetry and using the analog pain scale.

In Assessment 2, the legs of the participants were again measured by volumetry at 7:00 a.m. and the stockings were worn during the entire day with a further evaluation at 4:00 p.m. (volumetric and analog pain scale).

**Figure 1:**
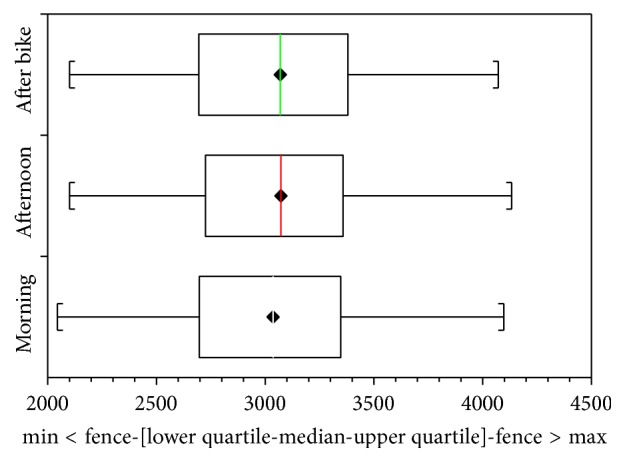
Interquartile variations in volume in the morning, the afternoon without using elastic stockings during the day, and after the treadmill session using stockings (Assessment 1).

**Figure 2:**
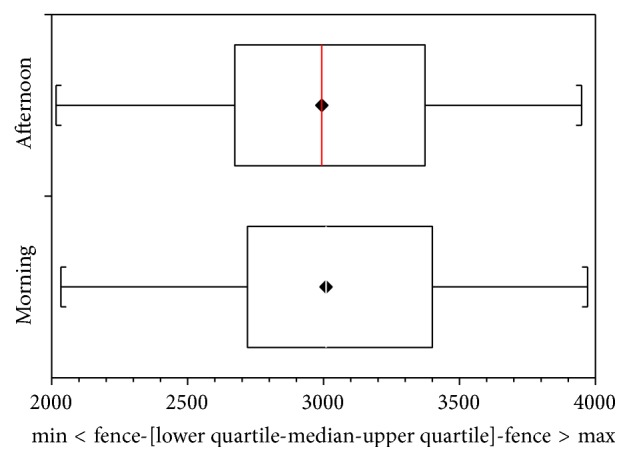
Interquartile variations in volume in the morning and the afternoon after using elastic stockings during the day (Assessment 2).

Consecutive patients were randomly assigned to two different groups where on the first day of the study one group followed Assessment 1 (Figure 1) and the other group followed Assessment 2 (Figure 2) and on the second day this was inverted.

The paired *t*-test and the Wilcoxon signed rank test were used for statistical analysis with an alpha error of 5% being considered acceptable.

The study was approved by the Research Ethics Committee of the Medicine School in Sao Jose do Rio Preto (FAMERP). The patients were provided with details of the study and those who chose to participate signed informed consent forms.

## 3. Results

Thirty-six of the legs were classified as CEAP C3 and six were CEAP C2.

On the day that the elastic stockings were only used during the treadmill session Assessment 1 (Figure 1), the mean volume of the legs in the morning was 3043.57 mL and by 4:00 p.m. this had increased to 3086.90 mL (paired *t*-test: *p* value < 0.0001). After walking for 50 minutes on the treadmill, the volume reduced to 3070.92 mL (paired *t*-test: *p* value < 0.03).

On the day that the elastic stockings were used during the entire day Assessment 2 (Figure 2), the initial mean volume was 3042.23 but this reduced to 3021.28 by 4:00 p.m. (paired *t*-test: *p* value < 0.0001).

In relation to pain, there was a reduction in pain after the treadmill session using the elastic stocking (Wilcoxon signed rank test: *p* value < 0.007) Table 1. The use of elastic stockings during the day also reduced the pain in the evening (Wilcoxon signed rank test: *p* value < 0.01).

## 4. Discussion

The current study evaluated patients with signs and symptoms of chronic venous disease classified as CEAP C3. It demonstrates that using elastic stockings during the entire day reduces the edema and pain. The pain was one of the most important symptoms in the evaluation of these patients because it is more uncomfortable than the edema itself. In the clinical practice, the edema is not always considered important; patients often neglect it, claiming that it is normal due to their daily activities.

The proposed treatment was the use of Venosan 20/30 knee-length elastic compression stockings which proved to be effective in reducing or controlling the edema and pain. However, stockings with higher compression ratings are recommended in more advanced cases of chronic venous disease.

The evaluation of the edema and pain showed that both these variables worsened during the day when stockings were not used but that they improved after wearing elastic stockings while walking on the treadmill. This opens a discussion about the possibility of more flexibility in the use of elastic stockings by patients. The best option is to use the stockings during the entire day; however, it has been shown that use for half the day is useful to reduce edema [5].

This new option, to use stockings in the evening when there is edema, has already been evaluated by the authors [9]. This study reinforces the reduction of edema by walking using elastic stockings in cases of CEAP C3 but also shows that this technique is effective in patients to reduce associated pain. It is important to note that it is essential to identify other possible causes of pain in the legs, especially orthopedic causes that may be compounded by walking.

This study was carried out in Brazil, a tropical country, and during the summer when the edema and other symptoms of venous and lymphatic diseases are aggravated the most. In the summer, there is more resistance to the use of elastic stockings by patients and so this flexibility may help to relieve the symptoms. The use of medications is another therapeutic option in these cases, but not every patient is adherent to drug treatment.

## 5. Conclusion

The reduction of edema and pain of the legs during the course of the day can be accomplished with the use of elastic stockings, as well as walking on a treadmill for 50 minutes in the afternoon while wearing elastic stockings.

## Figures and Tables

**Table 1:** Showing the pain scale values at the beginning of treatment (in the morning) and the afternoon after performing the bike.

Patients	Morning	Afternoon	After bike
1	0	0	0
2	0	0	0
3	3	5	3
4	0	2	0
5	2	3	4
6	0	0	0
7	0	0	0
8	0	0	0
9	0	0	0
10	0	0	0
11	0	0	0
12	3	3	3
13	6	7	0
14	3	0	0
15	2	3	0
16	2	4	0
17	1	4	1
18	0	4	0
19	0	4	1
20	2	2	4
21	3	3	0
